# A new model to identify node importance in complex networks based on DEMATEL method

**DOI:** 10.1038/s41598-021-02306-y

**Published:** 2021-11-24

**Authors:** Wentao Fan, Yuhuan He, Xiao Han, Yancheng Feng

**Affiliations:** 1 Department of Control and Systems Engineering, Nanjing University, Nanjing, 210093 China; 2 College of Biological Sciences and Technology, Beijing Forestry University, Beijing, 100083 China; 3 School of Cyberspace Science, Harbin Institute of Technology, Harbin, 150006 China; 4 School of Computer Science and Engineering, Beihang University, Beijing, 100191 China

**Keywords:** Applied physics, Computer science

## Abstract

It is still a hot research topic to identify node importance in complex networks. Recently many methods have been proposed to deal with this problem. However, most of the methods only focus on local or path information, they do not combine local and global information well. In this paper, a new model to identify node importance based on Decision-making Trial and Evaluation Laboratory (DEMATEL) is presented. DEMATEL method is based on graph theory which takes the global information into full consideration so that it can effectively identify the importance of one element in the whole complex system. Some experiments based on susceptible-infected (SI) model are used to compare the new model with other methods. The applications in three different networks illustrate the effectiveness of the new model.

## Introduction

Complex networks abstract complex systems in the real world into network structure diagrams and describe the relationships between components in the system through the edge-to-edge relationship between nodes. With the development in these years, complex networks have been applied to many fields such as finance, bionic algorithm, society, physics, quantum, resource allocation, social network, game theory, power grids, time series, fractal dimension, risk analysis and so on^1–7^. However, the importance of each node can’t be regarded as the same. For example, in an epidemic spreading network, we regard the node that has got high transmission capacity as the node with high importance. In a transportation network, a node connecting more cities must has higher importance than the nodes connecting less cities. So, how to effectively identify the node importance in different networks has received much attention.

Some methods to identify node importance have already been proposed such as degree centrality (DC)^8,9^ betweenness centrality (BC)^10,11^, closeness centrality (CC)^10^, eigenvector centrality (EC)^12^ and so on^13–16^. DC focuses on the degree of nodes. The degree describes the connections between the node and its surrounding neighbours. The more nodes a focal node is connected to, the more important this node is. Although DC is simple to calculate, the global information is ignored. BC calculates the times the node locates at the shortest paths among other two nodes. CC is defined as the inverse sum of the shortest distances to all other nodes from a focal node. BC and CC both take path information as a vital feature to identify node importance. While in large-scale networks, the computational complexity limits their application. Another shortcoming of BC is that the result obtained by BC will be 0 if few nodes locate at the shortest paths. And for CC, even small alteration in the structure of the networks will affect the order of nodes. EC applies normalized largest eigenvector to judge the node importance.

Some methods based on interaction between nodes also show good performances. The inverse-square law shows the intensity of the interaction between objects. Based on inverse-square law, a new method is proposed by Fei et al.^17^. It judges the node importance by calculating the interaction of one node between other nodes. The Gravity model^18^ considers both local information and path information. As the name implies, this model comes from the gravity calculation formula. In Gravity model, mass in gravity calculation formula is replaced by degree. Based on Gravity model, a weighted Gravity model is proposed by Liu et al.^19^. The weight of each node is calculated by the normalized largest eigenvector.^20^proposed that the location of principal eigenvector (PEV) and the delocalization of principal eigenvector will cause difficulties when ranking nodes based on EC which means that EC is not suitable for the networks with delocalized PEV. The weighted gravity model will face this problem, because the global information is gained from the normalized eigenvector.

DEMATEL^21,22^ method is often used to calculate the importance of an element in the entire system. Through the logical relationship between the elements in the system, DEMATEL can construct the direct relation matrix. The system elements’ degree of influence and degree of being influenced can be calculated by DEMATEL through matrix operations to obtain the importance of the elements in the whole system. More importantly, DEMATEL method not only deals with direct influence but also handles the indirect influence. Indirect influence only exists when there are three or more elements. Comparing nodes to planets, assume there are three planets $\alpha$, $\beta$, $\theta$. Regard the gravity between two planets as direct influence. There is not only gravity between planet $\alpha$ and planet $\beta$ and gravity between planet $\beta$ and planet $\theta$ but also the transitive gravity between planet $\alpha$ and planet $\theta$. The transitive gravity is called the indirect influence. Likewise, indirect influence also exists in networks. In this paper, a new model to identify node importance based on DEMATEL method is proposed. The interaction between two elements calculated by Gravity model is taken as the direct influence. That is, the larger the node’s degree and the shorter the distance between the nodes, the intensity of the interaction is larger. Based on DEMATEL method, the global information is fully considered.

The paper is organized as follows. “Preliminaries” section is the brief introduction of the preliminaries, reviewing some node centrality measures such as DC and BC. And the computing steps of DEMATEL method is also presented. The new model based on DEMATEL method is introduced in ″Proposed method” section. In ″Applications in real networks” section, the new model is applied in three real networks to test the effectiveness. In ″Conclusion” section, the conclusion of the whole work is presented.

## Preliminaries

### Centrality measures

A concrete network can be abstracted as $G=(V,E)$, where *V* is set of nodes and *E* denotes set of edges. Some node centrality measures such as DC and BC are introduced as follows:

#### Definition 1

degree centrality (DC). The centrality of node i denoted as $d_i$, can be obtained as^8^:

1 $$\begin{aligned} d_i = \sum _{j}^{N} x_{ij} \end{aligned}$$

Where *N* is the number of nodes in network and $x_{ij}$ represents the connection between node i and node j. If node i and node j is connected, then $x_{ij}$ equals to 1. Otherwise, $x_{ij}$ equals to 0.

#### Definition 2

betweenness centrality (BC) The centrality of node *i* denoted as $b_i$, can be calculated as^10,11^:

2 $$\begin{aligned} b_i = \sum _{m\ne i\ne n}^{N} \frac{p_{mn}(i)}{p_{mn}} \end{aligned}$$

Where *N* is the number of nodes and $p_{mn}$ is the number of shortest paths between node *m* and node *n*. $p_{mn}(i)$ represents the times that node i locates at the shortest paths between node *m* and node *n*.

#### Definition 3

closeness centrality (CC).

The centrality of node i denoted as *CC*(*i*), is defined as^10^:

3 $$\begin{aligned} CC(i) = \sum _{i}\left(\frac{x_{ij}}{N-1}\right)^{2}\left(\frac{1}{d_{ij}}\right) \end{aligned}$$

$x_{ij}$ is the connection between node i and node j and $d_{ij}$ is the distance between i and node j.

#### Definition 4

Eigenvector centrality (EC).

The centrality of node i denoted as *EC*(*i*), is defined as follow^12^:

4 $$\begin{aligned} EC(i) = \frac{1}{\lambda _{max}} \sum _{i=1}^{N}(x_{ij}e_{i}) \end{aligned}$$

Given a $N \times N$ matrix *M*, $\lambda _{max}$ is the largest eigenvalue of the *M*. *M* is the adjacency matrix of network *G*. $e_{i}$ is the ith element of the normalized largest eigenvector.

#### Definition 5

Gravity model The centrality of node i denoted as $CG_(i)$, is defined as follow^18^:

5 $$\begin{aligned} CG(i) = \sum _{i\ne j}^{N} \frac{k_{i}k_{j}}{(d_{ij})^2} \end{aligned}$$

where $k_i$ is the degree of node i and $d_{ij}$ is the distance between node i and node j. It referred to the formula for calculating gravity, in which mass is replaced by the degree. The Gravity model considers both local and path information. As the square of the distance increases,the gravity between the two celestial bodies is linearly attenuated.

#### Definition 6

Weighted Gravity model The centrality of node i denoted as *WG*(*i*), is defined as follow^19^:

6 $$\begin{aligned} WG(i) =e_{i} \times \sum _{i\ne j}^{N} \frac{k_{i}k_{j}}{(d_{ij})^2} \end{aligned}$$

$e_{i}$ is the ith element of the normalized eigenvector which is taken as the weight of each nodes.

### DEMATEL method

DEMATEL method^23,24^ which is based on graph theory is proposed by the Bottele Institute in USA. DEMATEL method is usually applied to calculate the importance of an element in the entire system. The main advantage of DEMATEL is that it not only considers direct influence but also considers indirect influence. The computing steps of DEMATEL is shown as follows:

Step 1 Construct the direct relation matrix:
  7 $$\begin{aligned} D_{n\times n}= \begin{pmatrix} 0&{}d_{12}&{}\cdots &{}d_{1n}\\ d_{21}&{}0&{}\cdots &{}d_{2n}\\ \vdots &{}\vdots &{}\ddots &{}\vdots \\ d_{n1}&{}d_{n2}&{}\cdots &{}0 \end{pmatrix} \end{aligned}$$
  The influencing factors of the system need to be determined in advance. $d_{ij}$ represents the direct influence between i and j.
Step 2 Normalize the direct relation matrix
  8 $$\begin{aligned} N_{n\times n}= \begin{pmatrix} 0&{}m_{12}&{}\cdots &{}m_{1n}\\ m_{21}&{}0&{}\cdots &{}m_{2n}\\ \vdots &{}\vdots &{}\ddots &{}\vdots \\ m_{n1}&{}m_{n2}&{}\cdots &{}0 \end{pmatrix} \end{aligned}$$
  where
  9 $$\begin{aligned} m_{ij}=\frac{d_{ij}}{max(\sum \nolimits _{j=1}^{n}d_{ij},\sum \nolimits _{i=1}^{n}d_{ij})} \end{aligned}$$
Step 3 Obtain total relation matrix based on normalized relation matrix.
  Total relation matrix T is obtained as follows:
  10 $$\begin{aligned} T=\mathop {lim} \limits _{n\rightarrow \infty }(N+N^2+N^3+...+N^{n-1}+N^{n}) \end{aligned}$$
  This process is the most significant part of DEMATEL method. The normalized relation matrix continues to multiply in order to add indirect influence between all elements.
Step 4 Obtain the causal parameters *R* and *C* based on total relation matrix
  11 $$\begin{aligned} R_i=\sum _{j} t_{ij}(j=1,2,3,\ldots ,n) \end{aligned}$$
  12 $$\begin{aligned} C_j=\sum _{i} t_{ij}(j=1,2,3,\ldots ,n) \end{aligned}$$
  *R* is used to represent the influence of each element and *C* is used to represent the degree of being influenced.
Step 5 Calculate the importance
  13 $$\begin{aligned} a_i=R_i+C_i \end{aligned}$$

## Proposed method

A wide variety of node centrality measurements have been proposed these years. Each has its own advantages and disadvantages. Whether the local and global information are fully utilized is the key point to judge the effectiveness of the method. The Gravity model considers both local and path information. In this article, the interaction between two nodes calculated by the Gravity model is taken as the direct influence. Based on DEMATEL method, the indirect influence in the network is also well addressed, making the new model more globally than the Gravity model. The specific steps of the new model is shown as follows:

Step 1 Construct the network Abstract complex systems in the real world into network structure as $G = (V, E)$. *V* is the set of nodes and *E* is the set of edges.
Step 2 Calculate the degree and the distance between nodes.
  In Gravity model, node degree represents local information and distance represents path information. The degree can be obtained as follows:
  14 $$\begin{aligned} k_i = \sum _{j}^{N} x_{ij} \end{aligned}$$
  where $x_{ij}$ is the connection between node i and node j. The distance is the length of the shortest paths between nodes.
Step 3 Generate the direct relation matrix
  15 $$\begin{aligned} D= \begin{pmatrix} 0&{}g_{12}&{}\cdots &{}g_{1n}\\ g_{21}&{}0&{}\cdots &{}g_{2n}\\ \vdots &{}\vdots &{}\ddots &{}\vdots \\ g_{n1}&{}g_{n2}&{}\cdots &{}0 \end{pmatrix} \end{aligned}$$
  $g_{ij}$ can be calculated as follows:
  16 $$\begin{aligned} g_{ij} = \frac{k_{i}k_{j}}{(d_{ij})^2} \end{aligned}$$
Step 4 Normalize the direct relation matrix
  17 $$\begin{aligned} N= \begin{pmatrix} 0&{}\grave{g_{12}}&{}\cdots &{}\grave{g_{1n}}\\ \grave{g_{21}}&{}0&{}\cdots &{}\grave{g_{2n}}\\ \vdots &{}\vdots &{}\ddots &{}\vdots \\ \grave{g_{n1}}&{}\grave{g_{n2}}&{}\cdots &{}0 \end{pmatrix} \end{aligned}$$
  where
  18 $$\begin{aligned} \grave{g_{ij}}=\frac{g_{ij}}{max(\sum \nolimits _{j=1}^{n}g_{ij},\sum \nolimits _{i=1}^{n}g_{ij})} \end{aligned}$$
Step 5 Calculate the total relation matrix.
  Given the normalized matrix, the total relation matrix can be obtained as follows:
  19 $$\begin{aligned} T= \begin{pmatrix} 0&{}t_{12}&{}\cdots &{}t_{1n}\\ t_{21}&{}0&{}\cdots &{}t_{2n}\\ \vdots &{}\vdots &{}\ddots &{}\vdots \\ t_{n1}&{}t_{n2}&{}\cdots &{}0 \end{pmatrix} \end{aligned}$$
  where
  20 $$\begin{aligned} t_{ij}=\mathop {lim} \limits _{n\rightarrow \infty }(\grave{g_{ij}}+(\grave{g_{ij}})^2+(\grave{g_{ij}})^3+...+(\grave{g_{ij}})^{n-1}+(\grave{g_{ij}})^{n}) \end{aligned}$$
  The normalized relation matrix continues to multiply in order to add indirect influence between all nodes in complex networks.
Step 6 Calculate the causal parameters *R* and *C* based on total relation matrix
  21 $$\begin{aligned} R_i=\sum _{j} t_{ij}(j=1,2,3,\ldots ,n) \end{aligned}$$
  22 $$\begin{aligned} C_j=\sum _{i} t_{ij}(j=1,2,3,\ldots ,n) \end{aligned}$$
Step 7 Calculate node importance *a*
  23 $$\begin{aligned} a=R+C \end{aligned}$$
  Add the influence of this one node on other nodes and the degree of being influenced of this node as the importance of this node in the whole network.

**Figure 1:**
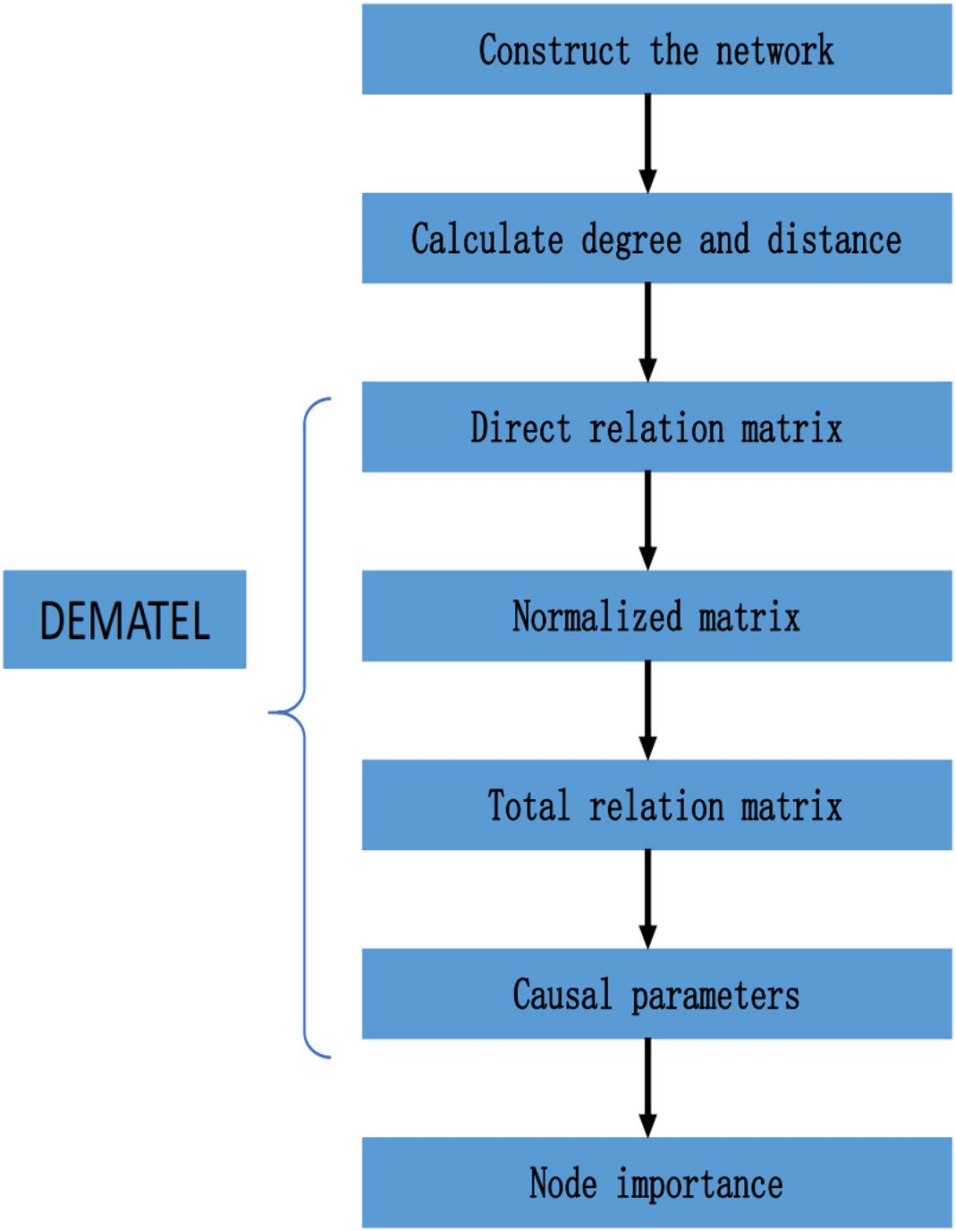
The flow chart of the new model.

The flow chart of the new model is illustrated in Fig. 1.

The new model is improved based on the gravity model from a more global perspective. The novelty is that the new model considers indirect influence among nodes based on DEMATEL method and takes it as global information to enhance the effectiveness. The new proposed method weighted gravity model is also improved based gravity model from a global perspective, but the global information is gained from the eigenvector of the adjacency matrix. The ideas of the two methods are completely different. The weighted gravity model will face the problem that networks with delocalized principal eigenvector will cause difficulties in assigning centrality weights to the nodes. However, the new model dose not has this problem.

## Applications in real networks

### DATA

In this section, three real networks are introduced to test the effectiveness of the new model. They are Jazz, NS and USAir. Jazz describes the network of Jazz musicians in USA. The node is the musician and the edge is the connection between two Jazz musicians. If the two musicians have ever played jazz together, then there is an edge between the two nodes. NS is the network of scientists in USA. The edge is the cooperation between two scientists. USAir describes American Airlines network diagram. The data can be downloaded from http://pajek.imfm.si/doku.php?id=data:pajek:vlado&s[]=air. The node is the airport and the edge is the air route between two airports. The basic characteristics of the networks are show in Table 1:

**Table 1:** Basic characteristics of the networks.

Networks	Jazz musicians	NS	USAir97
n	198	379	332
m	2472	914	2126
k	27.70	4.82	12.81
d	2.24	6.04	2.74

n denotes the number of nodes. m is the the number edges. k and d are average degree and average distance, respectively. The network diagrams are shown in Fig. 2. In addition, a certain analysis of degree distribution of each network is applied. The analysis includes two parts-the degree distribution of each node in the network and the degree probability distribution of the network which is shown in Figs. 3, 4 and 5.

**Figure 2:**
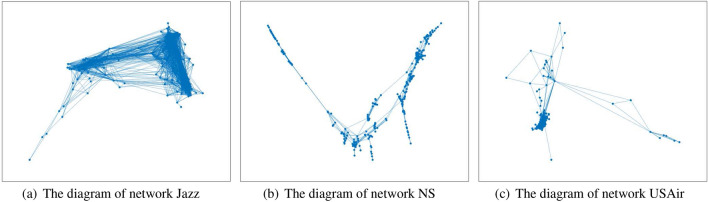
The network diagrams.

**Figure 3:**
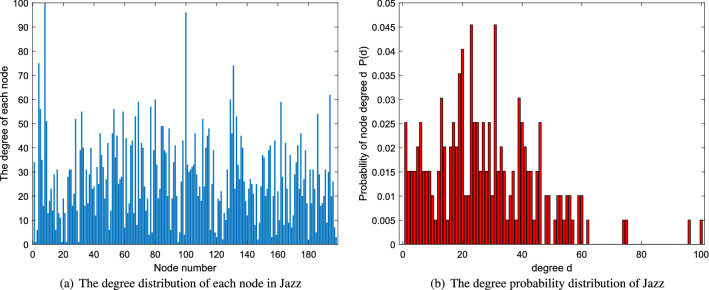
The degree distribution of network Jazz.

**Figure 4:**
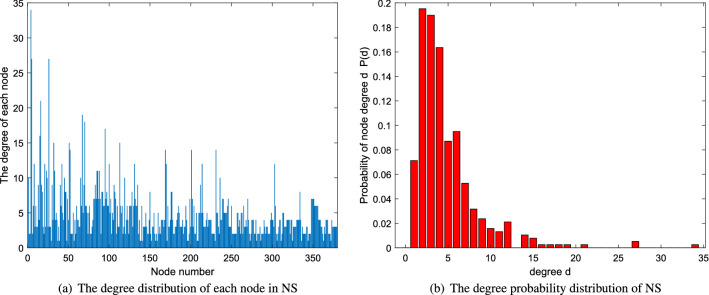
The degree distribution of network NS.

**Figure 5:**
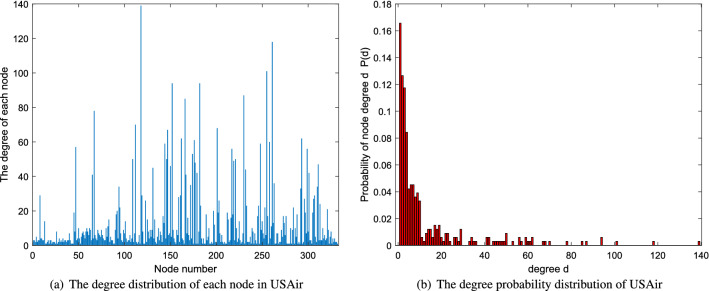
The degree distribution of network USAir.

### Centrality scores calculated by different methods

In this experiment, the new model is applied to calculate the top 10 important nodes in four different networks. As a comparison, the centrality measurements mentioned before such as BC, EC are used in the same networks. Nodes are arranged in descending order of their node degree. In this way, the top 10 nodes selected represent the nodes with high influence in the network. The results are shown in Tables 2, 3 and 4.

**Table 2:** The top 10 important nodes in Jazz.

BC	DC	CC	EC	Gravity	WGravity	DGravity
8	8	8	100	28	186	28
155	100	100	4	186	28	186
100	4	131	8	136	136	136
186	131	194	131	175	175	175
131	194	69	80	98	23	98
136	80	4	129	158	113	158
127	129	32	5	100	112	113
60	69	53	194	113	38	100
28	162	111	69	33	143	33
69	77	162	53	23	178	23

**Table 3:** The top 10 important nodes in NS.

BC	DC	CC	EC	Gravity	WGravity	DGravity
26	4	26	4	4	70	4
51	5	95	5	70	214	70
169	26	51	16	214	303	214
95	16	231	15	303	212	303
67	67	100	45	212	67	212
5	70	52	46	67	119	16
231	95	5	47	16	270	67
100	15	44	176	119	150	5
44	32	234	177	5	69	119
66	51	297	250	304	348	304

**Table 4:** The top 10 important nodes in USAir.

BC	DC	CC	EC	Gravity	WGravity	DGravity
118	118	118	118	152	13	221
8	261	261	261	221	8	144
261	255	67	255	230	144	13
201	152	255	182	144	65	152
47	182	201	152	261	221	230
182	230	182	230	182	47	65
255	166	47	112	166	45	166
152	67	166	67	13	313	8
313	112	248	166	255	142	182
13	201	112	147	201	296	47

**Figure 6:**
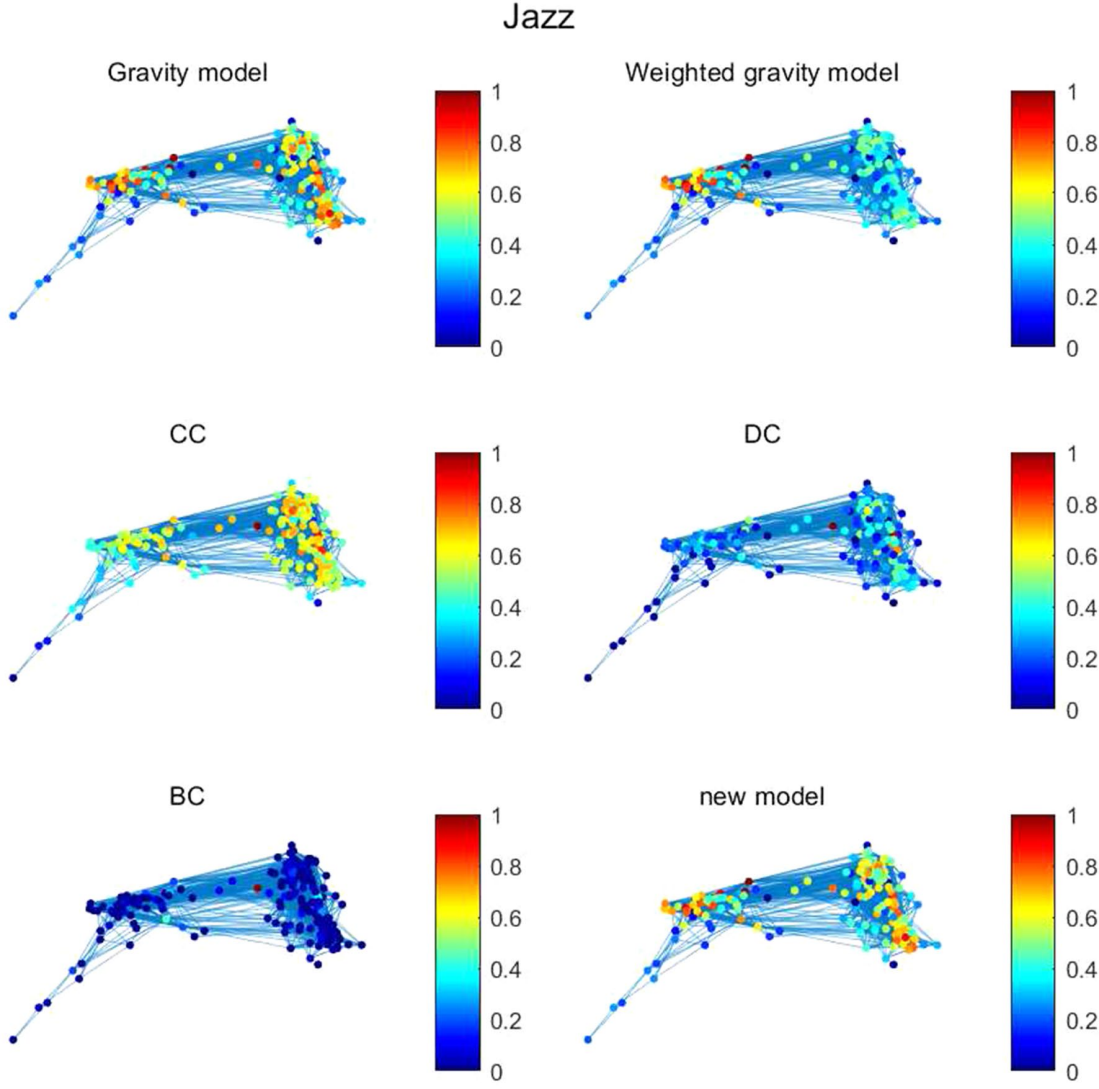
The figure describes node importance of six methods in Jazz. Gravity model, weighted gravity model and the new model basically perform the same. The value of nodes in BC is low.

**Figure 7:**
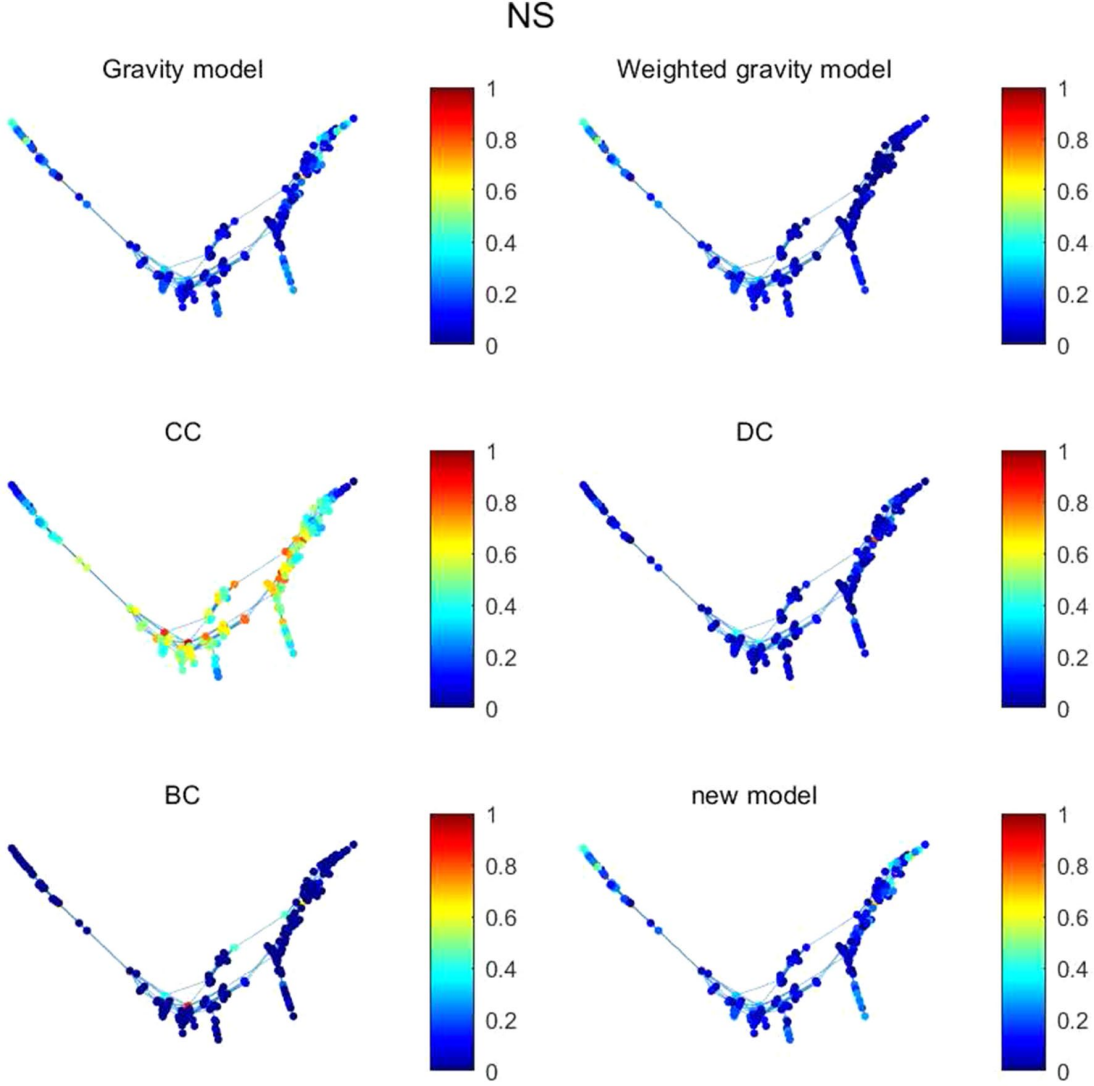
The figure describes node importance of six methods in NS. The value of each node in CC is much higher than other methods.

**Figure 8:**
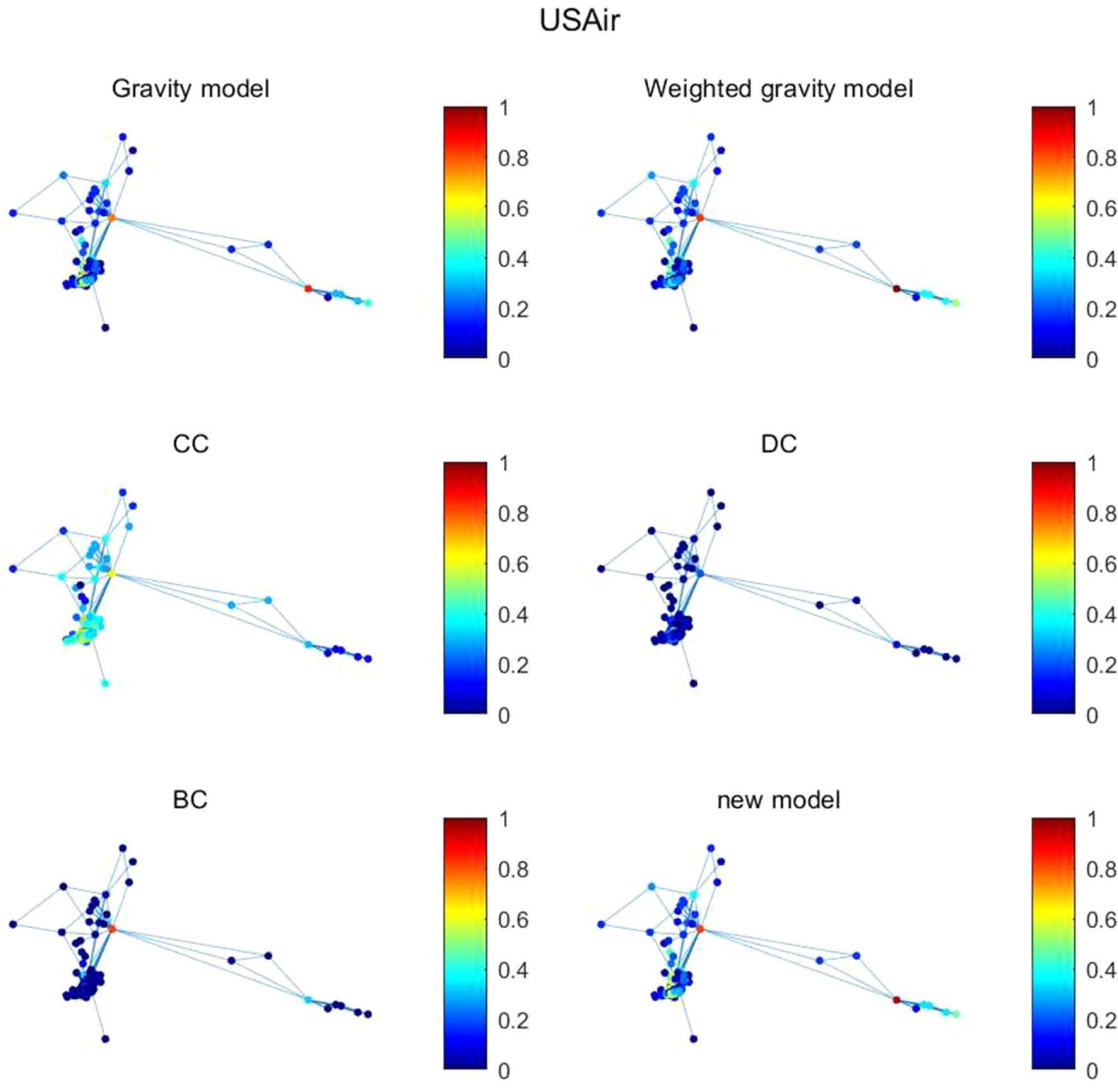
The figure describes node importance of six methods in USAir. The brighter the node color, the higher the node importance.

Since the new model is improved based on Gravity model, the new model has strong consistency with Gravity model. We can observe that in networks Jazz and NS, the top ten important nodes calculated by Gravity model and the new model are the same. Just the order of several nodes is different. In network Jazz, compared with BC, they both figure out node 28, node 186 and node 100. There are 6 same nodes between weighted gravity model and the new model. In network NS, the results between the new model and DC have the same 5 nodes in top ten nodes. The results between the new model and EC have got 3 same nodes. There are 6 same nodes between weighted gravity model and the new model. In network USAir97, the number of same nodes in top 10 important nodes are 5, 4, 3, 4, 7, 6 between the new model and BC, DC, CC, EC, Gravity model, weighted gravity model respectively. Through calculating top 10 important nodes in different networks, it can be observed that the new model has great consistency with Gravity model and weighted gravity model in identifying node importance. At the same time, the node importance calculation results are displayed in the form of a heat map Figs. 6, 7 and 8. In the network, the brighter the node color, the higher the node importance.

Subsequent experiment will continue to verify the consistency of the new model with other methods.

### Compare the correlation between the new model and other methods

**Figure 9:**
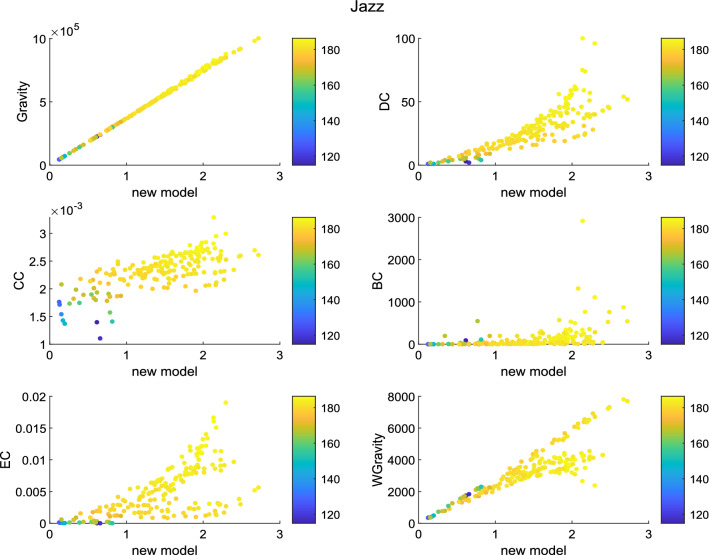
The figure describes the correlation between the new model and other methods in Jazz. The new model has a strong positive correlation with Gravity model. The liner relation with DC, EC and weighted gravity model is also clear.

**Figure 10:**
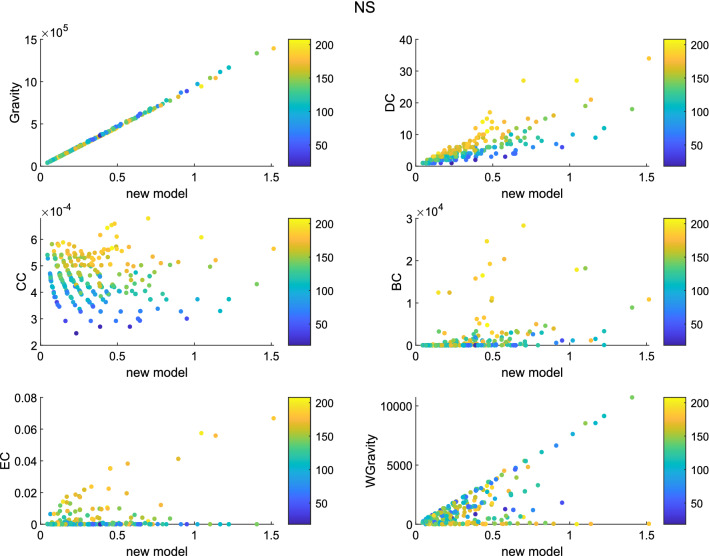
The figure describes the correlation between the new model and other methods in NS. Besides Gravity model and weighted gravity model, the new model shows positive correlation with DC.

**Figure 11:**
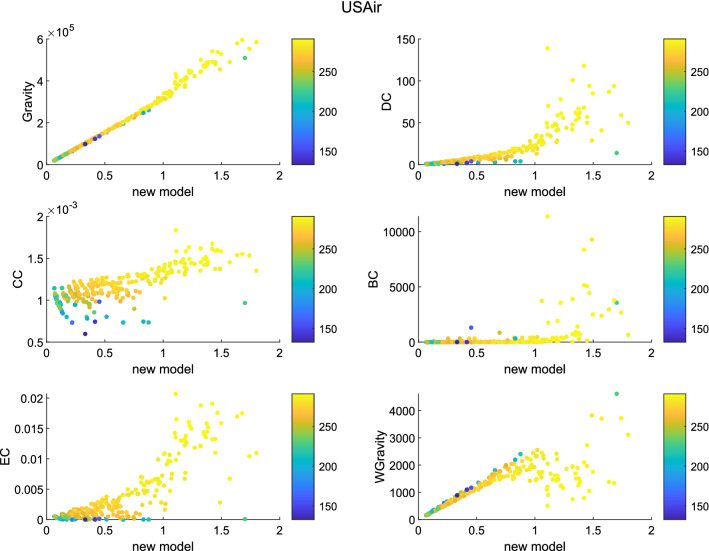
The figure describes the correlation between the new model and other methods in USAir. The new model shows strong relationship with Gravity model and weighted gravity model.

Before the experiment, the Susceptible-infected (SI) model^25–27^ has to be introduced. SI model is usually used to test the transmission capacity of nodes. In SI model, nodes have got two states : susceptible state and infected state. The infected node continuously infect other nodes with a specific probability $\beta$. We can identify node transmission capacity by calculating the number of nodes infected by a certain node within a period of time. The experiment results are shown in Figs. 9, 10 and 11.

Set the infection probability $\beta$ to 0.1. The number of experiments was set to 10 times. The experimental simulation time was set to 45. The color of the node represents its transmission capacity. The brighter the color, the stronger the transmission capacity. The ordinate and abscissa in the figures are both node centrality. Generally speaking, as centrality increases, the brighter the node in most graphs. It shows that the node sequence based on node centrality is consistent with the node sequence based on transmission capacity. According to figures, in network Jazz, the new model has a strong positive correlation with Gravity model. The liner relation with DC, EC and weighted gravity model is also clear. In network NS, besides Gravity model and weighted gravity model, the new model shows positive correlation with DC. In network USAir97, the new model shows strong relationship with Gravity model and weighted gravity model.

### Compare the effectiveness based on SI model

**Figure 12:**
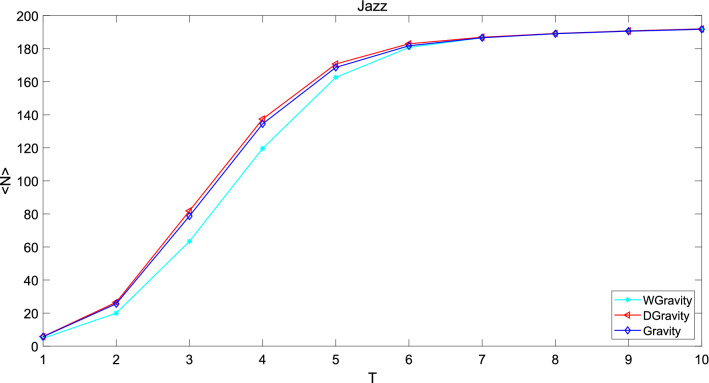
This figure compares the new model with Gravity model and weighted gravity model based on average infected nodes in network Jazz. Number of average infected nodes increases with time and eventually stabilizes. The growth rate decreases with time, and finally approaches to 0. The new model performs slightly better than Gravity model and much better than weighted gravity model. Because the number of average infected nodes of the new model is larger than that of Gravity model and weighted gravity model until it goes steadily which means that the top 50 nodes calculated by the new model are more important in the network.

**Figure 13:**
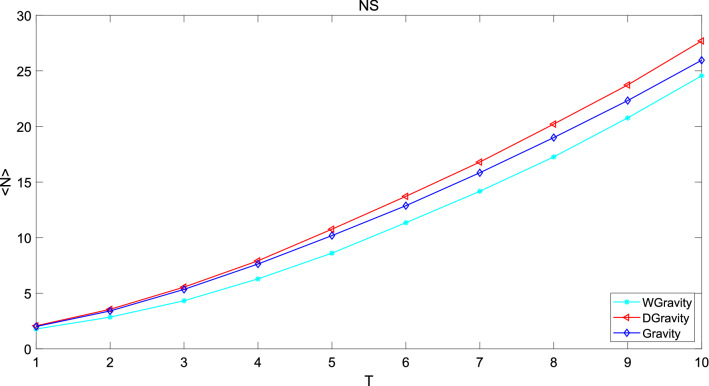
This figure compares the new model with Gravity model and weighted gravity model based on average infected nodes in network NS.The growth rate increase with time. Obviously, the new model is superior to Gravity model and weighted gravity model.

**Figure 14:**
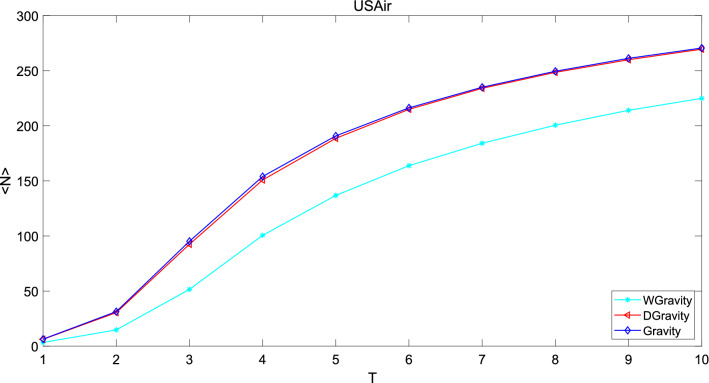
This figure compares the new model with Gravity model and weighted gravity model based on average infected nodes in network USAir. The new model performs slightly worse than Gravity model, but much better than weighted gravity model.

In SI model, the more nodes an infected node infects in the same time, the more important the node is. In this experiment, the number of average infected nodes is used to test the effectiveness of the new model. First, the nodes in the network are sorted by centrality calculated by the proposed method. Then we take the top 50 nodes as source of infection. Each node will spread n times with a probability of $\beta$ within experimental simulation time. First, we record the total number of nodes infected by each node. Then, we divide it by the number of experiments to get each node’s average infected nodes. In addition we add the average infected nodes of the top 50 nodes. In this experiment, probability of node infection was set to 0.1. The experimental simulation time was set to 10. The number of experiments was set to 10. The new model is compared with Gravity model and weighted gravity model. The experiment results are shown in Figs. 12, 13 and 14.

The abscissa of the figure is time and the ordinate is the average infected nodes. The larger the slope of the curve, the greater influence the selected nodes have. In network Jazz, number of average infected nodes increases with time and eventually stabilizes. The growth rate decreases with time, and finally approaches to 0. The new model performs slightly better than Gravity model and much better than weighted gravity model. Because the number of average infected nodes of the new model is larger than that of Gravity model and weighted gravity model until it goes steadily which means that the top 50 nodes calculated by the new model are more important in the network. In network NS, the growth rate increase with time. Obviously, the new model is superior to Gravity model and weighted gravity model. In network USAir97, the new model is slightly worse than Gravity model, but much better than weighted gravity model. In general, the new model consist with Gravity model but it is obviously superior to weighted gravity model. The superiority of the new model can be demonstrated in this experiment.

### Compare the correlation with SI model

Before the experiment, Kendall’s tau^28^ is introduced. Kendall’s tau is used to measure the consistency of two equal-length sequences. The more consistent the two sequences, the greater the value of tau. Assume there are two sequences *X* and *Y* of length n. $x_i$ and $y_i$ are the ith elements of *X* and *Y* respectively. Given two sequence pairs $(x_{i}, y_{i})$ and $(x_{j}, y_{j})$ , if $x_{i} > x_{j}$ and $y_{i} > y_{j}$ or $x_{i} < x_{j}$ and $y_{i} < y_{j}$ , the pairs are regarded as positive. Otherwise, they are regarded as negative.

**Figure 15:**
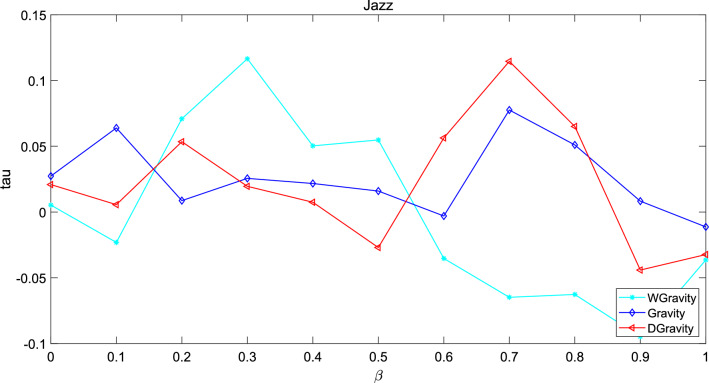
This figure describes the correlation between the methods based on gravity model and the standard sequence generated by SI in Jazz. The new model performs better when $\beta$ is in the interval 0.55–0.8. Gravity model shows the worst performance.

**Figure 16:**
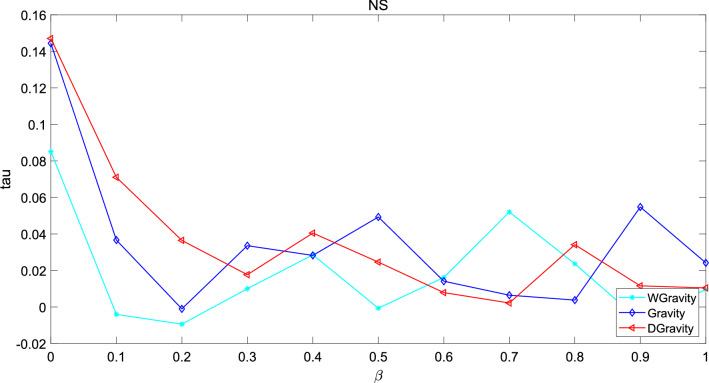
This figure describes the correlation between the methods based on gravity model and the standard sequence generated by SI in NS. The performance of the new model is better in the early stage, and the performance of the three methods is close to the same in the later stage.

**Figure 17:**
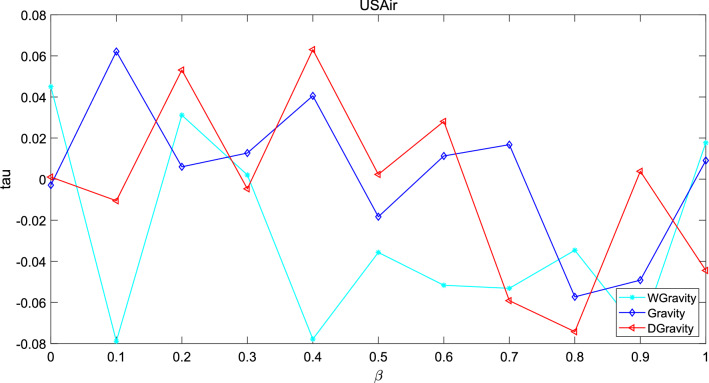
This figure describes the correlation between the methods based on gravity model and the standard sequence generated by SI in USAir. when $\beta$ > 0.5, the tau of the new model is below 0 which means the number of negative pairs is larger than the number of positive pairs.

The Kendall’s Tau is defined as:

24 $$\begin{aligned} tau=\frac{x_{p}-x_{n}}{n(n-1)} \end{aligned}$$

where $x_p$ denotes the positive sequence pair and $x_n$ denotes the negative sequence pair. n is the number of sequence pairs. First, the node sequence is ordered by the node centrality calculated by the new model, the Gravity model and weighted gravity model. In addition, another sequence is ordered by the transmission capacity calculated by SI model. Set infection probability $\beta$ from 0 to 1. Time was 20 and the experiment times was set to 100 times. The experiment results are shown in Figs. 15, 16 and 17.

These graphs describe the difference between the node sequence based on SI model and the sequence based on node centrality as $\beta$ increases. The greater the value of tau, the stronger the correlation between the two sequences. In network Jazz, the new model performs better when $\beta$ is in the interval 0.55–0.8. Gravity model shows the worst performance. In network NS, the performance of the new model is better in the early stage, and the performance of the three methods is close to the same in the later stage. In network USAir97, when $\beta$> 0.5, the tau of the new model is below 0 which means the number of negative pairs is larger than the number of positive pairs.

## Conclusion

Recently, how to identify node importance in networks has become a hot issue. Whether the local and global are fully combined is the key point to test the effectiveness of the method. This article proposed a new model based on DEMATEL method. The interaction between two nodes obtained by Gravity model is taken as the direct influence. Based on DEMATEL method, the indirect influence is fully considered making the model more globally than Gravity model. The limitation is that although each experiment is repeated many times, the results obtained by the experiments based on SI model will still have some randomness. In general, the results demonstrate the superiority of our method.
